# Short Fatigue-Crack Growth from Crack-like Defects under Completely Reversed Loading Predicted Based on Cyclic R-Curve

**DOI:** 10.3390/ma17184484

**Published:** 2024-09-12

**Authors:** Keisuke Tanaka, Yoshiaki Akiniwa

**Affiliations:** 1 School of Engineering, Nagoya University, 4-7-203 Ketsuzen-cho, Nishinomiya 662-0037, Japan; 2 Department of Mechanical Engineering and Materials Science, Yokohama National University, 79-5 Tokiwadai, Hodogaya-ku, Yokohama 240-8501, Japan; akiniwa-yoshiaki-xd@ynu.ac.jp

**Keywords:** short fatigue-crack, fatigue crack closure, fatigue growth threshold, fatigue limit, crack-like defect, modified strip-yield model

## Abstract

Understanding short fatigue-crack propagation behavior is inevitable in the defect-tolerant design of structures. Short cracks propagate differently from long cracks, and the amount of crack closure plays a key role in the propagation behavior of short cracks. In the present paper, the buildup of fatigue-crack closure due to plasticity with crack extension from crack-like defects is simulated with a modified strip yield model, which leaves plastic stretch in the wake of the advancing crack. Crack-like defects are assumed to be closure-free and do not close even under compression. The effect of the size of crack-like defects on the growth and arrest of short cracks was systematically investigated and the cyclic R-curve derived. The cyclic R-curve determined under constant amplitude loading of multiple specimens is confirmed to be independent of the initial defect length. Load-shedding and Δ*K*-constant loading tests are employed to extend the cyclic R-curve beyond the fatigue limit determined under constant amplitude loading. The initiation stage of cracks is taken into account in R-curves when applied to smooth specimens.

## 1. Introduction

In order to guarantee structural integrity, design engineers have been facing challenges in fatigue strength assessment of steel structural components, which usually contain imperfections. The determination of the tolerance level is a primary issue. Fatigue cracks always start at the spot of stress concentration, such as notches or defects, in structural components, and subsequent crack propagation results in the fracture of structures. Classical design of notch fatigue is based on the fatigue strength reduction factor, which is dependent on the size of the defects even if defects have the same stress concentration factor. When the defect size is very small, the fatigue strength of defected components is not reduced. On the other hand, as the size becomes larger, the fatigue strength is reduced by the amount of stress concentration factor or the stress intensity factor. There is an urgent need to establish a defect-resistant design methodology that takes defect size effects into account.

Understanding short fatigue-crack propagation behavior is integral to defect-tolerant design. The fatigue strength of components with small defects is determined by crack propagation, but not by crack initiation. In steel components subjected to stress amplitude below the fatigue limit, nonpropagating short cracks have been observed near defects [1,2,3]. Even in smooth specimens, nonpropagating small cracks have been observed below the fatigue limit [4,5,6,7,8], and the propagation condition controls the fatigue limit of smooth specimens.

Previously proposed models for predicting the initial deceleration or arrest of short cracks in notch fatigue are classified into two groups. One is based on the decrease of the amount of plasticity with distance from the notch tip, which induces crack deceleration or arrest as proposed by Smith et al. [9] and Haddad et al. [10]. The other is based on fatigue-crack closure. The buildup of crack closure with crack extension induces the decrease of the effective range of stress intensity factor (SIF), Δ*K*_eff_, resulting in deceleration or arrest of crack propagation. Tanaka et al. [11] showed that the Δ*K*_eff_ value was a controlling parameter for short crack growth in near-threshold regime of notch fatigue. Under the situation of excess notch plasticity, Nishikawa et al. [12] reported that all the growth data of short cracks were successfully correlated with long cracks using the Δ*K*_eff_ parameter, even though the amount of crack opening was enhanced in the notch-plastic zone.

For smooth specimen fatigue, Abdel-Raouf et al. [13] proposed that surface strain concentration was the main reason for nonpropagating cracks observed below the fatigue limit. Miller [14] proposed a microstructural barrier, such as a grain boundary, is the main cause for the arrest of small cracks. Akiniwa et al. [15] showed both microstructural barrier and crack closure could cause the nonpropagation of small cracks below the fatigue limit in smooth specimens.

To predict the fatigue threshold of components with notches or defects, Tanaka et al. [16] first proposed the cyclic R-curve method assuming the buildup of crack closure as the main mechanism of increase in resistance against crack growth. Cyclic R-curves are expressed as the relation between the threshold SIF range Δ*K*_th_ and the amount of crack extension Δ*a*, where Δ*K*_th_ is an increasing function of Δ*a*. The R-curve method was successfully applied to predict the effects of notch geometry and precrack length on fatigue thresholds [17,18]. Later, several investigators [19,20,21,22,23,24] used the concept of cyclic R-curve in order to explain their fatigue threshold results of components with defects. The state of the art of the cyclic R-curve method is described in recent reviews [25,26]. In relation to the R-curve method, Chapetti [27,28] proposed a different method to predict short crack growth behavior. He derived the relation between Δ*K*_th_ and crack length from the experimental data under the assumption that Δ*K*_th_ was a single-valued increasing function of the current length, independent of the initial size of crack-like defects or precracks. His proposal is not necessarily based on crack closure as a main mechanism of increasing material resistance to crack growth.

Few data on crack closure of short cracks have been published because experimental measurements are very hard to conduct. Contrary to experimental measurements, computer simulation of crack closure enables a systematic examination of the effects of defect size, yield stress, and applied stress on crack closure. Useful guidelines to examine experimental data will be provided by the simulation. Near the threshold in a nonaggressive environment, the main mechanisms of crack closure are plasticity-induced crack closure (PICC) and roughness-induced crack closure (RICC) [29]. With respect to RICC, several models have been proposed, but they are not yet available to predict quantitatively small crack growth behavior. On the other hand, for PICC, Budiansky and Hutchinson [30] proposed a strip yield model of Dugdale type [31] leaving residual stretches on crack wake faces and analyzed steady state growth of long cracks. Based on the modified strip yield model, Newman [32] developed a numerical simulation method to compute the development of PICC with crack extension. Toyosada et al. [33] included elastic deformation in Newman’s rigid plastic yield strips. A modified strip yield model is also applied to predict the crack propagation rate based on calculated Δ*K*_eff_ [32,33] or combined with the damage accumulation model [34]. 

In our preceding papers [35,36,37], a modified strip yield model was applied to fatigue-crack growth from crack-like defects or precracks with various sizes under completely reversed cyclic loading. Crack-like defects are called precracks in the present paper. The model was two-dimensional, and the stress state was plane stress. The precracks examined are two types: open precracks (Type I) and closed precracks (Type II). Open precracks are those made by cyclic compression precracking and may also represent material defects such as pores or drilled holes. The initial crack faces do not touch each other even under the maximum compression. Examples of closed precracks are natural cracks made by conventional fatigue, followed by annealing for residual stress relief. They may have no initial clearance between crack faces, so the crack faces may touch each other under compression. According to the results of our preceding paper [36], open precracks give lower opening stress than closed precracks under any stress amplitude, so the Δ*K*_eff_ value for open cracks is larger than that for closed cracks. The prediction of fatigue thresholds for open precracks is conservative, giving a lower fatigue limit and shorter fatigue life.

In the present paper, only PICC buildup from open precracks will be dealt with in order to obtain conservative estimation of steel structural components. The effect of defect size on fatigue thresholds is systematically examined. The cyclic R-curve is determined under constant amplitude loading of multiple specimens. Load-shedding and Δ*K*-constant loading tests are employed to extend the cyclic R-curve beyond the fatigue limit of precracked specimens determined under constant amplitude loading. In order to apply the R-curve method to smooth specimen fatigue, the crack initiation stage is taken into account in the R-curve method.

## 2. Simulation Procedure

A crack is extending straight from both ends of a crack-like defect or precrack in an infinite plate under a constant amplitude of completely reversed loading applied perpendicular to the defect. The assumed stress state is plane stress, and the loading mode of cracks is mode I. Plastic deformation ahead of crack tips is modeled by a yield strip, which is elastic perfectly plastic. The wake faces of advancing cracks have residual stretches, while the faces of precracks have no residual stretch. In the simulation, an initial clearance of 0.1 mm is attached to precracks to avoid crack face contact. The influence of the clearance of precracks on the stress distribution is not considered. For precracked specimens, the start of crack extension is called crack initiation in the present paper.

The simulation method used in the present paper is basically the same as the one used in the preceding papers [35,36]. The essence of the method is summarized in Appendix A.

Simulated materials are structural steels whose Young’s modulus is 206 GPa and Poisson’s ratio is 0.30. The yield stress is 400 MPa. The half-length of a crack-like defect ranges from 0.01 to 100 mm, such as 0.01, 0.03, 0.05, 0.1, 0.3, 1, 3, 10, 30, and 100 mm.

The threshold value of the effective SIF range Δ*K*_effth_ is adopted as the threshold condition for crack growth. The reported values of steels are about 2~3 MPa$\sqrt{m}$, independent of the material yield strength [11,16,17,18,25,26,38,39]. Using the unloading compliance method [40], Tanaka et al. measured the threshold range, Δ*K*_effth_, of short fatigue cracks of several steels in various situations and reported values around 3 MPa$\sqrt{m}\;$ [11,16,17,18,39]. They did not find any significant effect of *R* ratio on Δ*K*_effth_.

The threshold value of Δ*K*_th_ for long cracks at *R* ratios higher than 0.8 is expected to be equal to Δ*K*_effth_. The reported values of Δ*K*_th_ are between 2 and 3 MPa$\sqrt{m}$ for steels with the yield strength ranging from 300 to 1800 MPa [38,41]. Many investigators used compression precracking of notched specimens to produce closure-free precracks [19,20,22,23,24,25,26,42]. They interpreted the SIF range for crack initiation from precracks made by compression precracking as the intrinsic threshold value. For steels, their reported values are between 2 and 3 MPa$\sqrt{m}$, which are close to the critical SIF value for generation of cyclic plastic deformation at the crack tip [43,44].

On the basis of the above consideration, we adopt the threshold value of Δ*K*_effth_ = 3 MPa$\sqrt{m}$ as the crack growth criterion in the present paper.

## 3. Fatigue Growth Threshold of Short Cracks from Precracks

### 3.1. Buildup of Crack Closure with Crack Extension

In simulation, the crack opening stress, *σ*_op_, was determined as a function of the amount of crack extension from a precrack under *R* = −1. The relation between opening stress ratio *R*_op_ = *σ*_op_/*σ*_max_ and crack extension Δ*a* changes with the applied maximum stress, σ_max_, relative to the yield stress. The main results are summarized as follows. For open precracks, the opening stress at crack initiation σ_op_ is equal to *σ*_min_ and *R*_op_ = *σ*_op_/*σ*_max_ = *R* = −1. The opening stress initially shows a quick increase and then gradually approaches a steady state value as the crack extends longer. The steady state value of the opening stress ratio of long cracks decreases with increasing ratio of *σ*_max_ to the yield stress.

### 3.2. Growth and Arrest of Short Cracks

The effective range of SIF, Δ*K*_eff_, over which crack tip is open is given by

(1) $$\Delta K_{\mathrm{eff}}=K_{\max}-K_{\mathrm{op}}=K_{\max}\left(1-K_{\mathrm{op}}/K_{\max}\right)=\Delta K\left(1-R_{\mathrm{op}}\right)/\left(1-R\right)$$

where *K*_max_ is the maximum SIF, *K*_op_ is the crack-tip opening SIF, and *R*_op_ = *K*_op_/*K*_max_. The range of opening SIF, Δ*K*_op_, over which the crack tip is closed is expressed as

(2) $$\Delta K_{\mathrm{op}}=K_{\mathrm{op}}-K_{\min}=\left(\sigma_{\mathrm{op}}-\sigma_{\min}\right)\sqrt{\pi a}=\Delta \sigma_{\mathrm{op}}\sqrt{\pi a}=\Delta K-\Delta K_{\mathrm{eff}}$$

where $K_{\min}=\sigma_{\min}\sqrt{\pi a}$ is the minimum SIF. At crack initiation for open precracks, *a* = *a_i_*, *K*_op_ = *K*_min_ and Δ*K*_eff_ = Δ*K*_effth_. The stress range for crack initiation, Δ*σ_w_*_1_, is

(3) $$\Delta \sigma_{w1}=\Delta K_{\mathrm{effth}}/\sqrt{\pi a_{i}}$$

where *a_i_* is the initial defect size.

**Figure 1:**
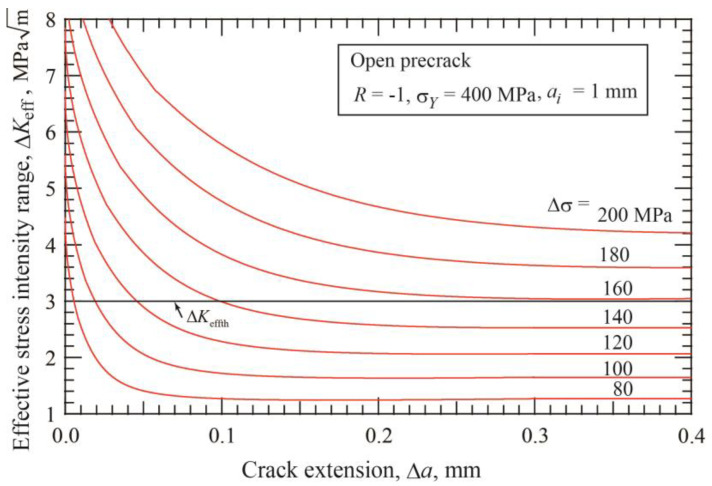
Change of Δ*K*_eff_ with crack extension under several applied stresses for precrack of 1 mm length (*σ_Y_* = 400 MPa, *R* = −1, open precrack).

After crack initiation under constant amplitude stress cycling, the applied stress intensity range, Δ*K*, increases with crack extension, and the effective stress intensity range, Δ*K*_eff_, initially decreases. Figure 1 shows the change in Δ*K*_eff_ with crack extension Δ*a* for an open precrack with the initial length of *a_i_* = 1 mm under seven stress ranges from 80 to 200 MPa with a step of 20 MPa. The crack is arrested when Δ*K*_eff_ drops below Δ*K*_effth_. The nonpropagating crack length Δ*a*_np_ becomes longer with increasing stress. For the case of *a_i_* = 1 mm, the fatigue limit for crack initiation is Δ*σ_w_*_1_ = 54 MPa, and that for fracture is Δσ*_w_*_2_ = 159 MPa.

**Figure 2:**
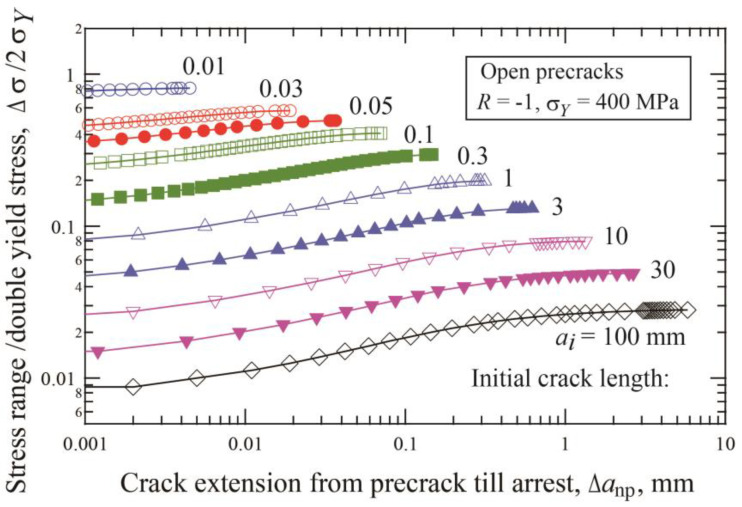
Change of crack extension until arrest with the applied stress range for precracks with various lengths (*σ_Y_* = 400 MPa, *R* = −1, open precrack).

**Figure 3:**
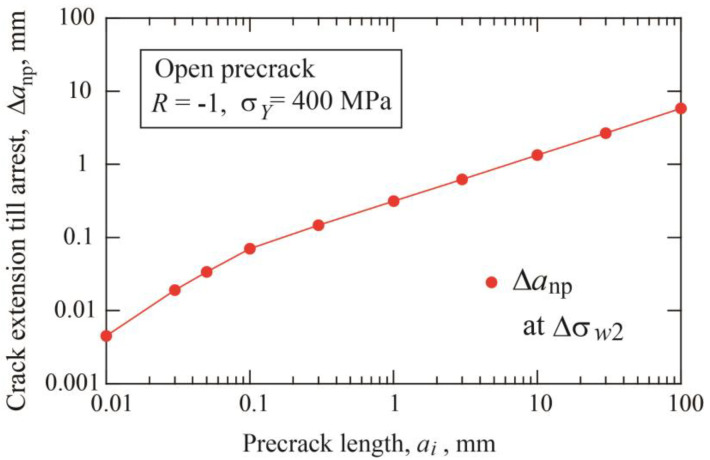
Crack extension until arrest, Δ*a*_np_, at Δσ*_w_*_2_ as a function of precrack length (*σ_Y_* = 400 MPa, *R* = −1, open precrack).

Figure 2 shows the change of the crack extension until arrest, Δ*a*_np_, with the applied stress range relative to double yield stress, Δ*σ*/2*σ*_Y_, for precracks with various lengths. For each precrack length, the data were obtained under constant amplitude loading of multiple specimens. The same results can be obtained by step-up amplitude loading of a single specimen [39]. For each precrack length, the stress range corresponding to the longest crack extension is the fatigue limit, Δ*σ_w_*_2_. Figure 3 indicates the change of the crack extension until arrest, Δa*_np_, at the fatigue limit Δ*σ_w_*_2_ with the precrack length. The Δa*_np_ length increases with increasing precrack length. Under step-up amplitude loading or constant amplitude loading, the nonpropagating crack longer than Δa*_np_ is not obtainable.

### 3.3. Effect of Precrack Length on Growth and Arrest of Short Cracks

**Figure 4:**
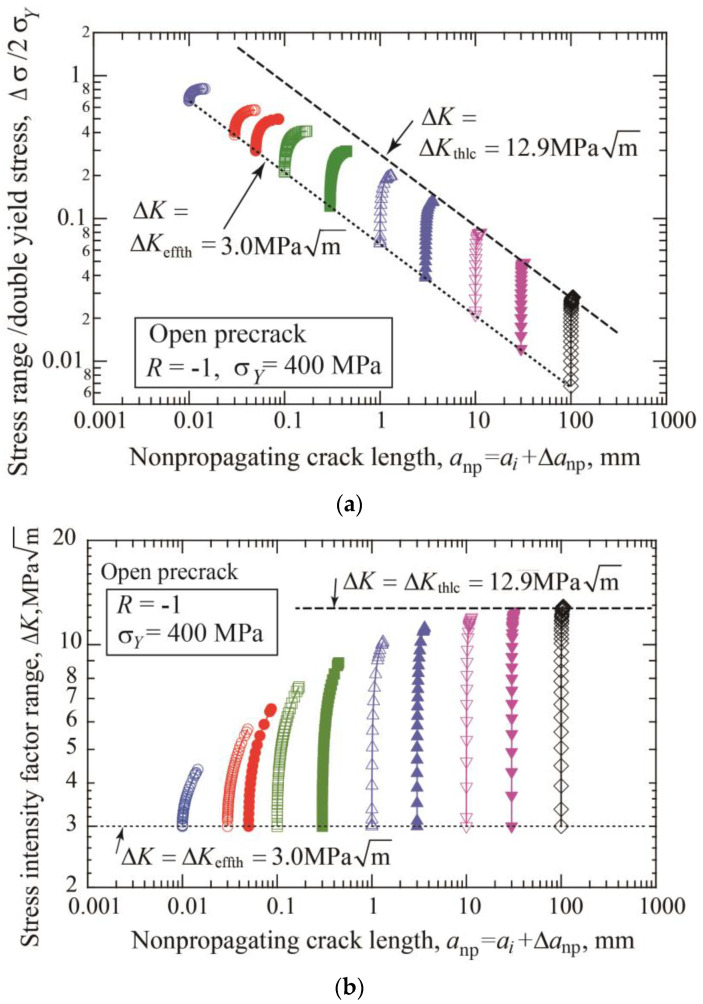
Change of nonpropagating crack length with stress range and SIF range for various precrack lengths from 0.01 to 100 mm (*σ_Y_* = 400 MPa, *R* = −1, open precrack). (**a**) Stress range/double yield stress vs. nonpropagating crack length. (**b**) Threshold SIF range vs. nonpropagating crack length.

The fatigue threshold diagram of specimens with precracks is shown in Figure 4a. The threshold stress range divided by double yield stress is plotted against the nonpropagating crack length, which is the sum of the initial precrack length plus crack extension until arrest. The dotted line connecting the crack initiation data indicates the crack initiation threshold given by Equation (3). The dashed line corresponds to the threshold SIF range for long cracks Δ*K*_thlc_ = 12.9 MPa$\sqrt{m}$. Figure 4b is a different presentation of the fatigue threshold diagram, where the threshold SIF range is plotted against the nonpropagating crack length. The horizontal dotted line indicates the crack initiation condition, Δ*K* = Δ*K*_effth_ = 3 MPa$\sqrt{m}$, and the dashed line means the threshold of Δ*K* = Δ*K*_thlc_ = 12.9 MPa$\sqrt{m}$. For each precrack length, the data points plotted between two lines correspond to crack extensions until self-arrest obtained under step-up amplitude loading. The Δ*K*_th_ at the fatigue limit for fracture is nearly constant for crack lengths above 10 mm and shows a tendency to decrease with shorter crack lengths.

### 3.4. Fatigue Threshold Diagram

**Figure 5:**
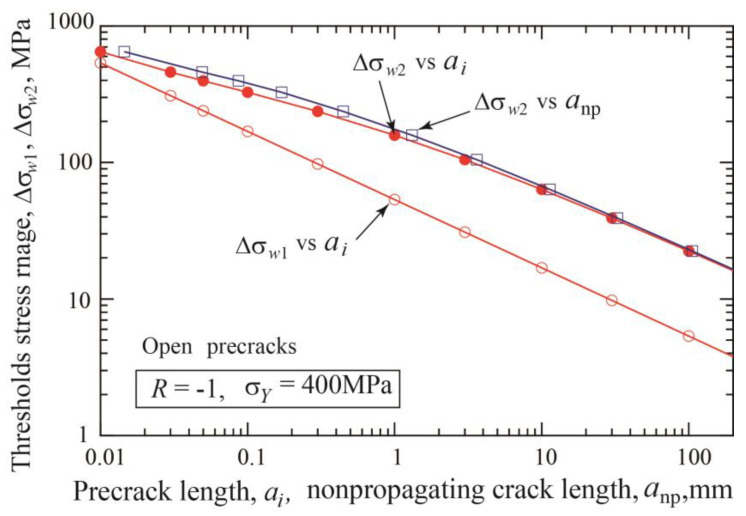
Changes in threshold stresses for crack initiation and fracture with crack length (*σ_Y_* = 400 MPa, *R* = −1, open precrack).

The fatigue threshold diagram of materials with defects is often called the Kitagawa–Takahashi diagram (K–T diagram), where the fatigue limit is in most cases plotted against the initial size of defects [45,46,47]. Likewise, the threshold stress ranges for crack initiation, Δ*σ_w_*_1_, and for fracture, Δ*σ_w_*_2_, obtained in the present simulation are plotted against the initial precrack length *a_i_* in Figure 5. Since the nonpropagating crack is formed, Δ*σ_w_*_2_ can be plotted at the nonpropagating crack length *a*_np,_ which is the sum of the precrack length plus the crack extension, *a*_np_ = *a_i_* + Δ*a*_np_. The data with the red circles are shifted horizontally to those with the blue squares by the amount of crack extension in Figure 5. Here, the former is called Type A plot and the latter Type B plot. The Δσ*_w_*_1_ value is given by Equation (3) and is the straight line in log-log diagram; there is no difference between Type A and B plots. The Δσ*_w_*_2_ value has a horizontal shift between Type A and Type B plots and bends horizontally at shorter crack lengths in both plots.

The threshold values of SIF are calculated from Δ*σ_w_*_1_ and Δ*σ_w_*_2_, and initial precrack length *a_i_* by the following formulae:

(4) $$\Delta K_{w1}=\Delta \sigma_{w1}\sqrt{\pi a_{i}}=\Delta K_{\mathrm{effth}}$$

(5) $$\Delta K_{w2}=\Delta \sigma_{w2}\sqrt{\pi a_{i}}$$

**Figure 6:**
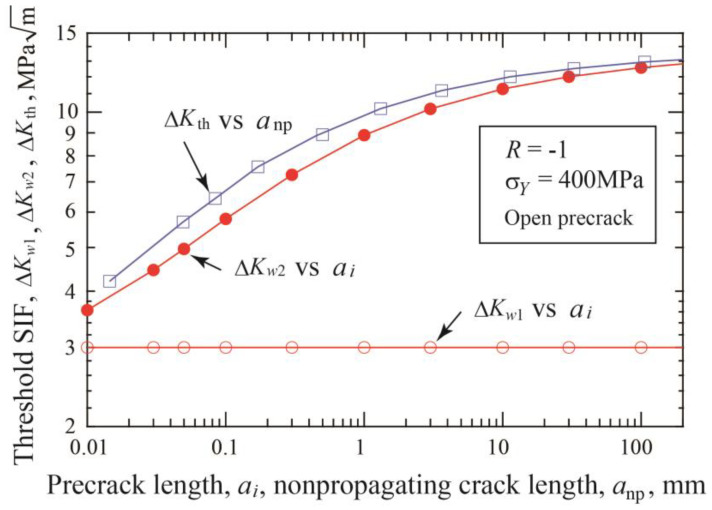
Changes in threshold SIF ranges for crack initiation and fracture with crack length (*σ_Y_* = 400 MPa, *R* = −1, open precrack).

Both SIF values are plotted against the initial precrack size, *a_i_*, with the open and closed red circles in Figure 6. Since crack extension takes place until arrest, the true SIF value, Δ*K*_th_, at the threshold is larger than the Δ*K_w_*_2_ value and is expressed by

(6) $$\Delta K_{\mathrm{th}}=\Delta \sigma_{w2}\sqrt{\pi \left(a_{i}+\Delta a_{\mathrm{np}}\right)}=\Delta \sigma_{w2}\sqrt{\pi a_{\mathrm{np}}}$$

where *a*_np_ is the nonpropagating crack length. In Figure 6, Δ*K*_th_ is plotted with the open squares against *a*_np_. The difference between Δ*K_w_*_2_ and Δ*K*_th_ is fairly large for short cracks and is much reduced for longer cracks.

In the K–T diagram, the threshold stress range, Δσ*_w_*_2_, is usually plotted against the initial precrack length, or defect size, neglecting the crack extension until arrest, as shown with the Type A plot in Figure 5. On the other hand, there is some confusion in the plot of the threshold SIF against the defect size in published reports. Kitagawa et al. [45] determined the threshold SIF using a load-decreasing test. The threshold value was plotted against the crack length at arrest, not at the initial crack length, which means Δ*K*_th_ plotted against *a*_np_ = *a_i_* + Δ*a*_np_. On the other hand, Murakami and Endo [48,49] calculated the threshold SIF from the initial defect area, which corresponds to Δ*K_w_*_2_ plotted against *a_i_*. The plot of Δ*K_w_*_2_ vs. *a_i_* is called Type A, and that of Δ*K*_th_ vs. *a*_np_ is Type B. It should be noted that there is a fairly large difference between Type A and B plots in the short-crack domain, as shown in Figure 6.

## 4. Cyclic R-Curve for Predicting Short Crack Growth

### 4.1. Cyclic R-Curve Determined by Constant Amplitude Loading of Precracked Specimens

The arrest of advancing fatigue cracks under constant loading is caused by the increase in material resistance against crack growth due to crack closure. The material resistance is expressed in terms of the threshold value Δ*K*_th_ and is an increasing function of the amount of crack extension Δ*a*_np_. This function was first named the cyclic R-curve by Tanaka and Akiniwa [16,17,18]. The threshold value, Δ*K*_th_, consists of the intrinsic component, Δ*K*_effth_, and the extrinsic component, Δ*K*_opth_, as follows:

Δ*K*_th_ = Δ*K*_effth_ + Δ*K*_opth_ (7)

The first term Δ*K*_effth_ is the threshold value of the effective SIF range and is assumed to be constant. The second term Δ*K*_opth_ is the opening SIF range at crack arrest, which is an increasing function Δ*a*_np_. Once the R-curve is established, the threshold stress for crack initiation and fracture for specimens with various notches or precracks can be determined from the comparison between the driving force curve and R-curve.

**Figure 7:**
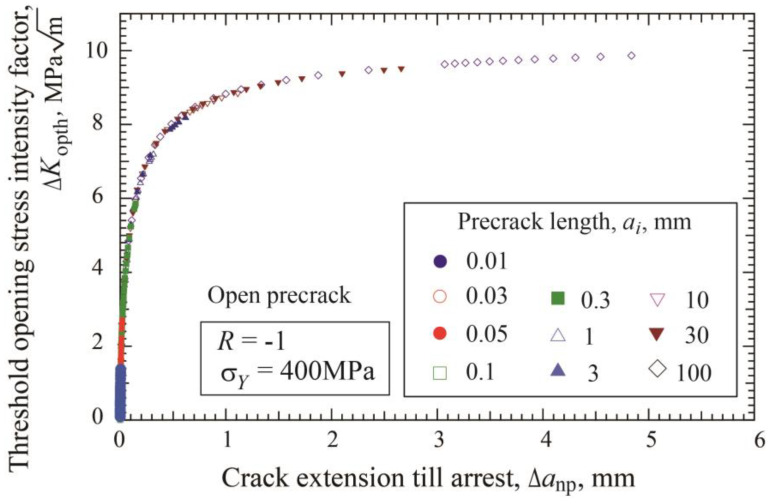
Effect of precrack length on the change of Δ*K*_opth_ with crack extension Δ*a*_np_ at the threshold (*σ_Y_* = 400 MPa, *R* = −1, open precrack).

The effect of precrack length on R-curve is examined. Since the increase of Δ*K*_th_ with crack extension is caused by Δ*K*_opth_, the following discussion is focused on the relation between Δ*K*_opth_ and Δ*a*_np_. Figure 7 shows the Δ*K*_opth_ - Δ*a*_np_ relation obtained for ten precrack lengths ranging from 0.01 to 100 mm for open precracks. The Δ*K*_opth_ value at Δ*a*_np_ = 0 is 0 and increases with crack extension, approaching a constant value. It is worth noting that the difference in the Δ*K*_opth_ vs. Δ*a*_np_ relation among various precrack lengths is very small. We can draw the conclusion that R-curve is an increasing function of the amount of crack extension and is not influenced by the initial precrack length.

The establishment of the functional form of the cyclic R-curve is important, which can help to determine the shape of the R-curve from a limited amount of data. The following equation was used by McEvily et al. [21] and others [22,23,24,25,26]:

(8) $$\Delta K_{\mathrm{opth}}/\Delta K_{\mathrm{opthlc}}=1-\exp\left[-\left(\Delta a_{\mathrm{np}}/\Delta a_{1}\right)\right]$$

The following two equations were proposed by Akiniwa and Tanaka [35,36]:

(9) $$\Delta K_{\mathrm{opth}}/\Delta K_{\mathrm{opthlc}}=1-\exp\left[-\sqrt{\left(\Delta a_{\mathrm{np}}/\Delta a_{2}\right)}\right]$$

(10) $$\Delta K_{\mathrm{opth}}/\Delta K_{\mathrm{opthlc}}=\sqrt{\Delta a_{\mathrm{np}}/\left(\Delta a_{\mathrm{np}}+\Delta a_{3}\right)}$$

In the above equations, Δ*a*_1_, Δ*a*_2_ and Δ*a*_3_ are fitting parameters and indicate the extent of the short crack region. All three equations show the transition of Δ*K*_opth_ from 0 to Δ*K*_opthlc_ as the crack extension Δ*a*_np_ becomes longer. In our previous papers [35,36], it was concluded that Equations (9) and (10) were better fitting formulae than Equation (8) for R-curve. Examples of fitting parameters determined from the simulation data are summarized in Table 1. The values of Δ*a*_2_ and Δ*a*_3_ are about the same for the cases of the initial crack length *a_i_* = 100 and 1 mm and also equal to those for the other crack lengths. On the other hand, there is a fairly large difference in Δ*a*_1_.

### 4.2. Extension of R-Curve for Longer Crack Region

R-curves shown in Figure 7 are determined from constant amplitude loading tests of multiple specimens subjected to different stress amplitudes. R-curve data are also obtainable using step-up amplitude loading of a single specimen, and there is no difference between two R-curves determined by different methods [37]. For each precrack length, the longest crack extension until arrest, Δa*_np_, is obtained at the fatigue limit, Δσ*_w_*_2_. Therefore, in order to extend the R-curve beyond Δa*_np_, load-shedding or Δ*K*-constant loading tests should be adapted as reported by Pourheidar et al. [24]. In simulation, a precracked specimen is first subjected to constant amplitude loading until a nonpropagating crack is formed. Then, the applied stress amplitude is increased to a higher stress, followed by the load-shedding procedure until the crack is arrested again. According to ASTM E 647-00 standard [50], the normalized *K*-gradient, *C*, in load-shedding tests needs to be

(11) $$C=\left(\frac{1}{K}\right)\left(\frac{dK}{da}\right)\geq -\;0.08\;{\mathrm{mm}}^{-1}$$

when *C* = 0, Δ*K* is maintained constant. In simulation, the stress is decreased in each cycle of crack extension, following *C* = −0.08 mm^−1^ in load-shedding or *C* = 0 in Δ*K*-constant loading tests.

**Figure 8:**
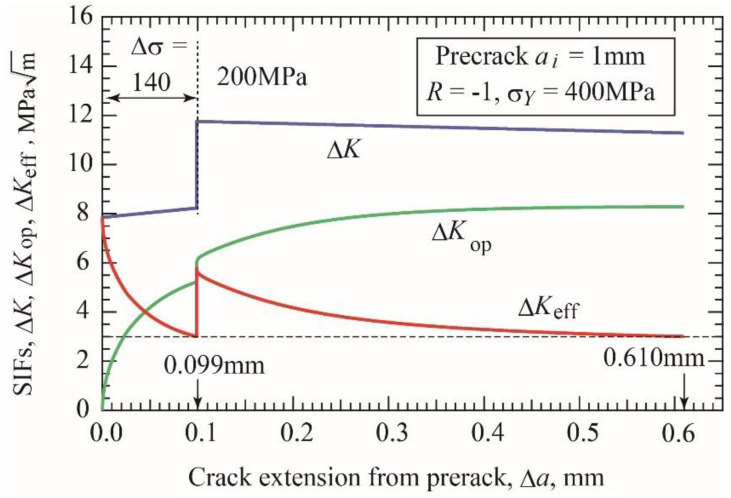
Changes in stress intensity factors Δ*K*, Δ*K*_op_, and Δ*K*_eff_, in load-shedding test for open precrack with length of 1 mm (*σ_Y_* = 400 MPa, *R* = −1).

The R-curve for the case of the precrack length of 1 mm is taken as an example next. Under constant amplitude loading, the fatigue limit for crack initiation is Δσ*_w_*_1_ = 54 MPa, and the fatigue limit for fracture is Δσ*_w_*_2_ = 159 MPa, accompanied by the nonpropagating crack extension of length Δa*_np_ = 0.314 mm. Figure 8 shows an example of load-shedding test. Under Δσ = 140 MPa, the crack extends 0.099 mm and is arrested at Δ*K* = 8.23 MPa$\sqrt{m}$ and Δ*K*_effth_ = 3 MPa$\sqrt{m}$. After obtaining the nonpropagating crack of Δ*a* = 0.099 mm, the load-shedding test is started at the stress level of 200 MPa and continued until crack arrest. With crack extension, the applied Δ*K* is decreased following Equation (11), and Δ*K*_eff_ also decreases because of buildup of Δ*K*_op_ as shown in Figure 8. The crack is arrested at the crack extension of Δ*a*_np_ = 0.610 mm in this example. After obtaining the nonpropagating crack extension of 0.099 mm under Δσ = 140 MPa, similar load-shedding tests are started at several different stress levels between 160 and 300 MPa and continued until crack arrest. Using the specimens with nonpropagating crack extension of 0.099 mm under Δσ = 140 MPa, Δ*K*-constant tests are also carried out at four levels: Δ*K* = 11, 11.5, 12, and 12.5 MPa$\sqrt{m}$ until crack arrest.

**Figure 9:**
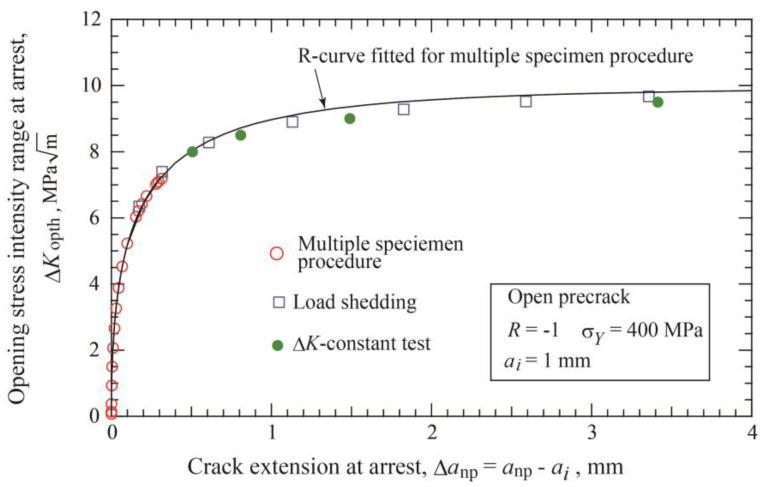
R-curve determined by constant-amplitude loading, load-shedding, and Δ*K*-constant loading tests for open precrack with *a_i_* = 1 mm (*σ_Y_* = 400 MPa, *R* = −1).

Figure 9 shows the relation between Δ*K*_opth_ and crack extension, Δ*a*_np_ = *a*_np_−*a_i_*, obtained by load-shedding and Δ*K*-constant tests, together with the results of constant amplitude loading of multiple specimens. The line represents the fitted relation of Equation (9) with the parameter given in Table 1. Both data obtained by load-shedding and Δ*K*-constant loading are plotted at longer nonpropagating cracks and above the limit of the data by constant amplitude loading indicated with the red circles. The data of load-shedding and Δ*K*-constant tests are fairly close to the fitted line in the whole region. The similar result of nice fitting was observed for the cases of the other precrack lengths. Therefore, it can be concluded that the relation of Equation (9) fitted for the data of constant amplitude loading tests of multiple specimens is applicable for crack extension longer than Δa*_np_.

**Figure 10:**
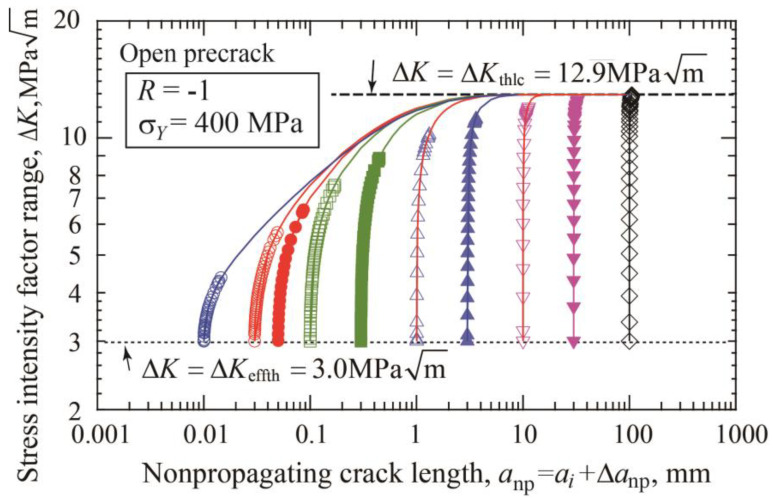
Changes in threshold stress intensity factor with nonpropagating crack length for open precracks. R-curves are fitted by Equation (9) (*σ_Y_* = 400 MPa, *R* = −1).

For open precracks, fitting parameters Δ*a*_2_ and Δ*a*_3_ in Equations (9) and (10) are rather independent of the precrack lengths. Using Δ*a*_2_ = 0.186 mm, the fitting curves of Equation (9) are drawn in Figure 10 together with the data of constant amplitude loading presented in Figure 4b. As explained in Figure 2 and Figure 3, the final data points for each precrack length correspond to the data at the fatigue limit Δ*σ_w_*_2_ under constant amplitude loading. Fitting curves are extended beyond the fatigue limit with the solid lines. Published data for the K–T diagram are obtained in various ways, including constant amplitude loading, load-shedding, and Δ*K*-constant tests using various precrack lengths. It should be noted that the present simulation clearly indicates that the reported data may scatter depending on the test method. Thorough examination of the test methods of published data is necessary together with the preparation method of cracks.

For constant amplitude loading, the connection of the final data points of each precrack length in Figure 10 gives the variation of the maximum value of Δ*K*_th_ with crack length. The relation between Δ*K*_th_ and *a*_np_ for the shortest precrack of 0.01 mm is the upper bound of the Δ*K*_th_ vs. *a*_np_ relation. In the case of the crack initiation stage included in R-curve, the upper bound relation is obtained for the case of the initiation crack length *a*_1_ = 0.022 mm as described in the next section (refer Equation (14)).

### 4.3. Inclusion of Crack Initiation Stage in R-Curve

It is well known that the K–T diagram of threshold stress range vs. precrack length can be divided into three regions [28,45,46,47,51]. In Region 1 of very short precracks whose length is less than *a*_1_, the precracks are nondamaging, without reducing the fatigue limit. For Region 3 above *a*_2_, the constant Δ*K*_th_ value is applicable for the crack growth threshold. In between Region 1 and 3, Region 2 is the micro crack region. In the preceding sections, we deal with short crack behavior in Region 2 based on the buildup of crack closure. For precracks longer than *a*_1_, there is no crack initiation stage; therefore, Δ*K*_opth_-curve expressions of Equations (9) and (10) are directly applicable. When applied to precracks less than *a*_1_, the present analysis should be modified. In those cases, fatigue fracture is caused by naturally formed cracks but not by existing precracks. The fatigue limit of smooth specimens is controlled not by crack initiation but by crack propagation [4,5,6,7,8]. The fatigue limit for crack initiation is less than the fatigue limit for fracture, Δ*σ_w_*_0_, and is here denoted by Δ*σ_w_*_01_.

The fatigue limit of steels with the yield strength of 400 MPa can be estimated as follows. The yield stress of steels, *σ_Y_*, is closely related to the ferrite grain size *d*. Etou et al. [52] proposed the following Hall-Pech relation between *σ_Y_* [MPa] and *d* [m] for ferritic steels for the range of yield stress from 100 to 700 MPa:

(12) $$\sigma_{Y}=100+0.600/\sqrt{d}$$

From this equation, the grain size of the present steel with σ*_Y_* = 400 MPa is estimated to be 4 μm. Next, the fatigue limit of smooth specimens may be obtainable from the following Hall-Petch relation between the fatigue limit Δσ*_w_*_0_ [MPa] and the grain size *d* [m] reported by Tachibana et al. [7] for steel WELL-TEN 60:

(13) $$\Delta \sigma_{w0}=373+0.267/\sqrt{d}$$

The estimated fatigue limit is 506 MPa.

In order to apply the R-curve method to smooth specimens, the crack initiation stage should be taken into account. In our previous study of notch fatigue [16], the crack initiation stage took place first, and then the crack propagation stage followed. In these cases, we assumed that the crack nucleated at the notch root was closure-free, and crack closure was built up as a function of the amount of extension from the nucleated crack. When nucleated cracks are open cracks, the R-curve with the fitting function of Equation (9) is modified to

(14) $$\Delta K_{\mathrm{th}}=\Delta K_{\mathrm{effth}}+\left(\Delta K_{\mathrm{thlc}}-\Delta K_{\mathrm{effth}}\right)\left\{1-\exp\left[-\sqrt{\left(a-a_{1}\right)/\Delta a_{2}}\right]\right\}$$

where *a*_1_ is the initiation crack length and Δ*a*_2_ = 0.186 mm.

**Figure 11:**
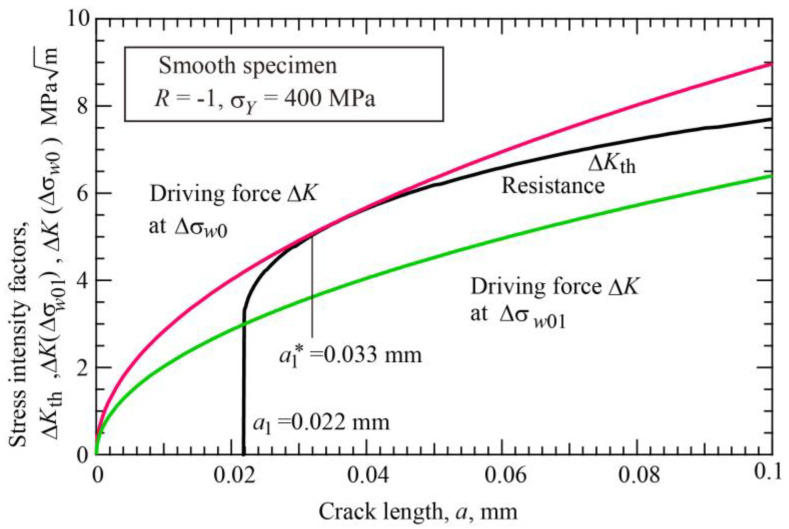
R-curve method for determination of thresholds of smooth specimens (*σ_Y_* = 400 MPa, *R* = −1).

Using Equation (14) and the fatigue limit Δσ*_w_*_0_ = 506 MPa, the initiation crack length *a*_1_ can be determined as follows. The necessary data for Equation (14) are given in Table 1. Figure 11 shows the R-curve method applied to smooth specimens to determine the *a*_1_ length. The red line is the driving force corresponding to Δ*σ_w_*_0_, and the black line is the R-curve, Equation (14). From the condition that the driving force is the tangent of the R-curve, the *a*_1_ length is determined as 0.022 mm. In this diagram, the green line crossing the point of the *a*_1_ and Δ*K*_effth_ corresponds to the crack initiation limit of smooth specimens, Δ*σ_w_*_01_, and is given by

(15) $$\Delta \sigma_{w01}=\Delta K_{\mathrm{effth}}/\sqrt{\pi a_{1}}$$

The calculated value is Δ*σ_w_*_01_ = 361 MPa. The nonpropagating crack at Δ*σ_w_*_0_ has the length at the contact point of the driving force and R-curve in Figure 11, i.e., *a*_1_* = 0.033 mm. The amount of crack extension from the initial length to the final length is 0.011 mm. The initiation crack length *a*_1_ determined is 5.5 times the grain size. Zerbst and Madia [23] determined the initiation crack size in a similar way for S355NL steel with the yield stress of 373 MPa. Their initial crack length is *a*_1_ = 0.035 mm, and the nonpropagating crack length is *a*_1_* = 0.05 mm, which are roughly the same order.

**Figure 12:**
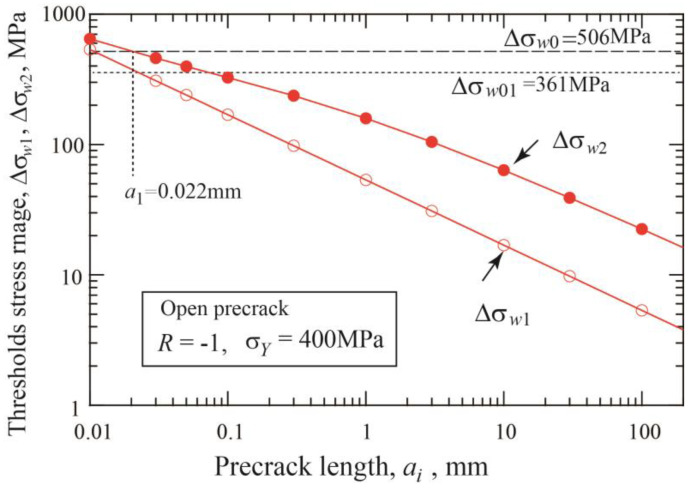
Diagram of fatigue limits for crack initiation and fracture against precrack size in order to determine the crack initiation length of smooth specimens (*σ_Y_* = 400 MPa, *R* = −1, Type A plot).

The same results can be obtained from the K–T diagram of the threshold stress range against the initial crack length (Type A plot) as shown in Figure 12. The horizontal line of Δ*σ_w_*_0_ = 506 MPa crosses the line of Δ*σ_w_*_2_ vs. *a_i_* relation at the crack length 0.022 mm, which is the initiation crack length *a*_1_. The vertical line at crack length *a*_1_ crosses the crack initiation line of Δ*σ_w_*_1_ vs. *a_i_* relation at 361 MPa, which corresponds to the fatigue limit for crack initiation Δ*σ_w_*_01_ of smooth specimens. 

The ratio of Δ*σ_w_*_01_ to the fatigue limit Δ*σ_w_*_0_ is 0.72. Many researchers reported that the crack initiation limit was less than the fatigue limit for fracture of smooth specimens of steels [4,6,8]. Nakazawa et al. [4] reported that the ratio of Δ*σ_w_*_01_ to the fatigue limit Δ*σ_w_*_0_ was around 0.76 to 0.79, while Yamada et al. [6] reported that the ratio was about 0.9, and Tanaka et al. [8] reported 0.79. The simulated ratio of 0.72 is slightly below the experimental data, and the low ratio may result from the neglection of crack closure of naturally initiated *a*_1_-sized cracks as described below.

Based on the microscopic observations near the fatigue threshold of smooth specimens of steels, Stage I shear cracks are normally formed along slip bands within a favorably oriented grain situated on the surface. After crossing the grain boundary, the crack gradually turns the growth direction along the plane perpendicular to the stress axis, often leaving a zigzag-shaped crack path. Nakazawa et al. [4] and Yamada et al. [5,6,7] reported that the fatigue limit of steel smooth specimens is controlled by the onset of Stage II propagation from shear mode or zigzag-shaped Stage I cracks. The maximum size of nonpropagating cracks observed below the fatigue limit of ferritic steels, Δ*σ_w_*_0_, ranges from a few grain sizes to an order of magnitude larger than the grain size depending on the microstructure [4,5,6,7,8]. Yamada et al. [5,6,7] suggested that large nonpropagating cracks below the fatigue limit were formed by connecting small shear cracks made in individual grains. The propagation of microstructurally small cracks can be blocked by the microstructural barriers, such as grain boundaries and phase boundaries, as proposed by Miller [14]. Akiniwa et al. showed both microstructural barrier and crack closure could cause the nonpropagation of small cracks below the fatigue limit of smooth specimens [15]. The present simulation is based on continuum mechanical analysis of crack closure development and does not take the microstructure into account. We interpreted the initiation crack length *a*_1_ in R-curves as the length at the transition from the microstructurally to the mechanically/physically short cracks as explained by Zerbst and Madia [23]. The initiation crack size is longer than the microstructural size or grain size, where the crack begins to show isotropic mechanical properties. At the same time, crack closure begins to develop. We conclude that the fatigue limit of smooth specimens is determined by the continuation of Stage II crack (or mechanically/physically small cracks) propagation, and the condition of growth or arrest of cracks is judged by using the R-curve method.

The above discussion is focused on Type I open precracks, but naturally initiated cracks may not be fully open. Stage I shear cracks may have a zigzag shape and have RICC. The initiated crack of length *a*_1_ may not be closure-free. In this case, the crack initiation condition shown in Equation (15) should be modified to include the contribution of crack closure using the effective fraction *U,* as follows:

(16) $$\Delta \sigma_{w01}=\frac{\Delta K_{\mathrm{effth}}}{U\sqrt{\pi a_{1}}}$$

Since *U* is less than 1, the ratio of Δσ*_w_*_01_/Δσ*_w_*_0_ becomes larger than 0.72, approaching experimental values. Subsequent development of crack closure is also influenced by the type of nucleated cracks. Further research is required for precise comparison between simulation results and experimental data.

Chapetti [27,28] proposed a method different from the R-curve method described above. He derived R-curve on the basis of the relations between Δ*K*_th_ and crack length *a* in the K–T diagram reported by several investigators. He assumed that the Δ*K*_th_ was a single-valued function of the current crack length, *a*, and independent of precrack length. He proposed the following formula:

(17) $$\Delta K_{\mathrm{th}}=\Delta K_{\mathrm{dR}}+\left(\Delta K_{\mathrm{thlc}}-\Delta K_{\mathrm{dR}}\right)\left\{1-\exp\left[-k\left(a-d\right)\right]\right\}$$

where *d* is the grain size, Δ*K*_dR_ is the microstructural threshold, *k* is a fitting parameter. The fatigue limit of smooth specimens, Δ*σ_w_*_0_, is related to Δ*K*_dR_ as follows:

(18) $$\Delta K_{\mathrm{dR}}=Y\Delta \sigma_{w0}\sqrt{\pi d}$$

where *Y* is the geometrical correction factor and is 0.65 for a semi-circular crack. For *k*, they proposed the following estimate:

(19) $$k=\frac{1}{4d}\frac{\Delta K_{\mathrm{dR}}}{\Delta K_{\mathrm{thlc}}-\Delta K_{\mathrm{dR}}}$$

Table 2 summarizes the data required for calculation of Equation (17), where Δ*a*_1_ = 1/*k*.

**Figure 13:**
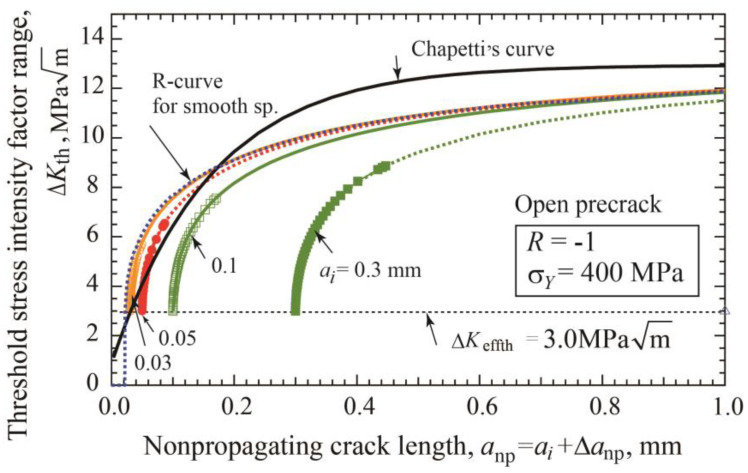
Relation between the threshold stress intensity and the nonpropagating crack length for smooth and precracked specimens (*σ_Y_* = 400 MPa, *R* = −1, open precrack).

Figure 13 shows the change of Δ*K*_th_ with crack length *a* plotted in linear scale diagram. R-curves for smooth specimens of Equation (14) and Chapetti’s curve of Equation (17) are drawn together with R-curves of specimens with precrack lengths of 0.03, 0.05, 0.1, and 0.5 mm. The R-curve of smooth specimens gives the upper bound of the other R-curves obtained for various lengths by simulation. Chapetti’s curve seems to be rather close to the R-curve for smooth specimens, but there is still a fairly large difference. It should be noted that there is one significant difference between the present R-curve method and Chapetti’s method. Chapetti claimed that the relation, Equation (17), was always applicable to predict the threshold Δ*K*_th_ value using the total crack length *a*, independent of the initial precrack length. When the crack starts in smooth specimens, there is not much difference between Chapetti’s equation, Equation (17), and our equation, Equation (14). On the other hand, when the crack started from crack-like defects or closure-free precracks, the Δ*K*_th_ is a single-valued function of amount of crack extension and not of the total crack length, as shown in Figure 10 and Figure 13 according to the present R-curve method. Further experimental examination would be necessary to resolve this contradiction.

## 5. Concluding Remarks

The buildup of PICC with crack extension from crack-like defects or precracks is simulated using a modified strip yield model under completely reversed loading. Closure-free precracks are assumed, having an initial clearance to remain open even under compression. The main results are summarized as follows:

(1) At crack initiation, the crack-tip opening stress is equal to the applied minimum stress and increases sharply with crack extension. The effective range of stress intensity factor Δ*K*_eff_ first drops and then changes to increase. Under the assumption of the threshold value of Δ*K*_eff_ for steels as Δ*K*_effth_ = 3 MPa$\sqrt{m}$, the crack is arrested when *ΔK*_eff_ drops below Δ*K*_effth_.
(2) There are two fatigue limits: Δσw1 is for crack initiation and Δσw2 is for fracture. As the precrack length becomes longer, the amount of extension of the onpropagating crack at Δσw2 becomes larger and the threshold SIF range, ΔKth, approaches a steady state value for long cracks ΔKthlc.
(3) The threshold SIF range ΔKth consists of two components: ΔKeffth and ΔKopth, where ΔKeffth is constant and ΔKopth is an increasing function of crack extension Δanp. The relation between ΔKth and Δanp, called cyclic R-curve, is independent of the initial precrack length. Two fitting functions proposed for R-curves will be useful to determine R-curves from limited experimental data.
(4) Load-shedding or ΔK-constant loading tests can be adapted to extend the cyclic R-curve beyond the fatigue limit of a precracked specimen under constant amplitude loading. For a given precrack length, the relation between ΔKth and the crack extension Δa is unique, following the fitted functions.
(5) For precracks shorter than the initiation crack length a1, the crack initiation stage of forming a crack length of a1 should be taken into account in R-curves. The length of the initiation crack, a1, is determined from the fatigue limit of smooth specimens, Δσw0, and the R-curve having the crack initiation stage.
(6) The propagation of the initiation crack of length a1 controls the fatigue limit of smooth specimens. The initiation crack length may correspond to the length at the transition from microstructurally short cracks to mechanically/physically short cracks and is several times larger than the grain size.

## Figures and Tables

**Table 1:** Fitting parameters for R-curves for open precracks with the initial lengths of 1 and 100 mm (*σ_Y_* = 400 MPa, *R* = −1).

Precrack Length *a_i_* [mm]	Threshold Δ*K*_op_ for Long Crack Δ*K*_opthlc_ $\left[\mathrm{MPa}\sqrt{m}\right]$	Threshold Δ*K*_eff_ Δ*K*_effth_ $\left[\mathrm{MPa}\sqrt{m}\right]$	Threshold Δ*K*_th_ for Long Crack Δ*K*_thlc_ $\left[\mathrm{MPa}\sqrt{m}\right]$	Parameter in Equation (8) Δ*a*_1_ [mm]	Parameter in Equation (9) Δ*a*_2_ [mm]	Parameter in Equation (10) Δ*a*_3_ [mm]
100	9.94	3.00	12.94	0.157	0.186	0.253
1	9.94	3.00	12.94	0.172	0.184	0.266

**Table 2:** Material properties for R-curve of steels with crack-like defects (*σ_Y_* = 400 MPa, *R* = −1).

Grain Size *d* [mm]	Fatigue Limit Smooth Spec. (Fracture) Δ*σ_w_*_0_ [MPa]	Fatigue Limit Smooth Spec. (Crack Init.) Δ*σ_w_*_01_ [MPa]	Crack Initiation Length *a*_1_ [mm]	Fitting Parameter in Equation (14) Δ*a*_2_ [mm]	Mi-cro-Threshold Δ*K*_dR_ $\left[mPa\sqrt{m}\right]$	Fitting Parameter in Equation (17) *k* [1/mm]	Fitting Parameter Δ*a*_1_ = 1/*k* [mm]
0.004	506	361	0.022	0.186	1.17	6.19	0.162

## Data Availability

The original contributions presented in the study are included in the article; further inquiries can be directed to the corresponding authors.

## Appendix A. Simulation of PICC Buildup Using a Modified Strip-Yield Model

In the simulation, we assume the plane stress state and the material is perfectly plastic without work hardening. In strip yielding at the maximum stress, the crack wake and the plastic zone are divided by a finite number of elements. The plastic zone ahead of the crack tip is divided into 40 elements, where the division is denser near the precrack tip. A precrack is also divided into 20 elements with uneven width, where the division is denser near the precrack tip. The details of the computational procedure are presented in our preceding papers [34,35]. They are listed as follows:

(i) Lumping of elements in the crack wake is carried out in order to suppress the increase of the number of elements due to crack extension.
(ii) Under the maximum stress, *σ*_max_, the crack opening displacement (COD) is determined as the sum of CODs due to the applied stress and to the yield stress, *σ_Y_*, in the plastic zone.
(iii) During unloading to the minimum stress, *σ*_min_, the stress in the plastic zone is reversed to yield in compression, and the crack faces start to contact. As described above, the initial clearance is assumed for open (Type I) precrack surfaces. The stresses in the plastic zone and on the contacted crack wake are determined by solving the simultaneous linear equations for element stresses with the Gauss-Seidel method. The element stress, *σ_i_*, is modified as follows: (a) For the whole region, if *σ_i_* < −*σ_Y_*, then *σ_i_* = −*σ_Y_*. (b) For elements in the crack wake, if *σ_i_* > 0, then *σ_i_* = 0. (c) For elements in the plastic zone, if *σ_i_* > *σ_Y_*, then *σ_i_* = *σ_Y_*, because the material is assumed to be perfectly plastic.
(iv) The crack-tip opening stress, *σ*_op_, is determined from the contact stress on the crack face.
(v) At the minimum stress, the crack is extended by d*a*, and one element of size d*a* is added behind the crack tip. The amount of crack extension in one cycle of simulation is determined as 0.2% of the maximum plastic zone size.
(vi) After crack extension, the above process of computation is repeated until the crack reaches a prescribed length or is arrested because of increasing crack closure.
